# Robotic‐Assisted Extended Thymus Carcinoma Resection With Unilateral Total Pleurectomy and Hyperthermic Intrathoracic Chemotherapy (HITOC)

**DOI:** 10.1155/cris/5591698

**Published:** 2026-08-26

**Authors:** Maria Andrea Kröplin, Peter Keller, Nicolas Eckstein, Hruy Menghesha, Philipp Feodorovici, Jens Buermann, Joachim Schmidt, Donatas Zalepugas

**Affiliations:** ^1^ Department of General, Visceral, Thoracic and Vascular Surgery, University Hospital Bonn, Bonn, Germany, uni-bonn.de; ^2^ Department of Thoracic Surgery, Helios Hospital Bonn/Rhein-Sieg, Bonn, Germany

**Keywords:** case report, HITOC, RATS, SCC, thymus carcinoma

## Abstract

Thymic squamous cell carcinoma (SCC) is an aggressive form of thymic cancer that is often diagnosed at an advanced stage. Hyperthermic intrathoracic chemotherapy (HITOC) is a treatment option for advanced thoracic and especially pleural malignancies, though it is not yet the standard of care. However, the success of treating pleural cancers with HITOC protocols has been well documented in selected cases. Robotic‐assisted thoracoscopic surgery (RATS) has become the standard procedure for thymectomy, even for locally advanced tumors. In this report, we describe the treatment of a 53‐year‐old patient with a locally advanced Masaoka–Koga stage IVa thymic SCC and concomitant limited pleural carcinomatosis who was successfully treated by RATS thymectomy and HITOC. The report highlights the diagnostic challenges, therapeutic approach, and postoperative care, emphasizing the advantages of minimally invasive, robot‐assisted surgery even in advanced cases. RATS with HITOC can be a safe and effective method for the treatment of advanced thymic carcinoma. This case report demonstrates that this procedure enables rapid postoperative recovery and can be performed with minimal morbidity.

## 1. Introduction

Squamous cell carcinoma (SCC) of the thymus is a rare but aggressive form of thymic malignancy. Thymic carcinomas account for around 0.2%–1.5% of all tumors and are often only diagnosed at an advanced stage. The incidence of thymic carcinoma is around 0.15 cases per 100,000 people per year, with the disease occurring more frequently in men than in women and being typically diagnosed between the ages of 40 and 60 [1, 2].

The diagnosis of thymic carcinoma is often incidental to imaging procedures performed for other reasons. Symptoms, if present, are nonspecific and may include chest pain, cough, shortness of breath, or systemic symptoms such as weight loss and night sweats. Diagnostic imaging, including CT and MRI, plays a crucial role in identifying and characterizing mediastinal masses. If thymic carcinoma is suspected, a fine needle biopsy or CT‐guided puncture is usually performed to determine the diagnosis histologically [1, 3].

The treatment of thymic carcinomas involves a multimodal approach that can combine surgery, radiotherapy, and chemotherapy. Surgical resection is the primary treatment option and aims to remove the tumor completely. However, in advanced stages (Masaoka–Koaga stage III), neoadjuvant chemotherapy is the standard of care. Hyperthermic intrathoracic chemotherapy (HITOC) is an innovative treatment option for patients with pleural metastases (stage IVa) used in combination with surgical cytoreduction. During HITOC, elevated temperatures are intended to increase the effectiveness of chemotherapeutic agents to eliminate remaining tumor cells after surgical cytoreduction (usually pleurectomy) [1, 4, 5]. Recent evidence further supports cytoreductive surgery combined with HITOC as a feasible treatment option in selected patients with thymic neoplasia and pleural involvement. A recent systematic review and single‐arm meta‐analysis reported low operative and overall mortality as well as low rates of acute kidney injury while also emphasizing that recurrence remains a relevant challenge for long‐term disease control [6].

Significant postoperative morbidity and mortality, as well as prolonged recovery after HITOC, are well known. Furthermore, it is important to note that HITOC is not a standard practice in the care of advanced thoracic cancers. However, in selected cases, such as young, otherwise healthy patients, this procedure may be considered an individualized therapeutic approach [7]. In this case report, we describe the treatment of a 53‐year‐old patient with SCC of the thymus and concomitant pleural carcinomatosis who was successfully treated with robotic‐assisted thoracoscopic extended thymectomy combined with extended robotic‐assisted thoracoscopic surgery (RATS)–pleurectomy and minimally invasive HITOC. The report highlights the individualized therapeutic approach, the operative procedure, and the postoperative care, with a focus on the advantages of minimally invasive, robot‐assisted procedures in extended resections.

## 2. Case Presentation

### 2.1. Anamnesis and Preoperative Course

**Figure 1:**
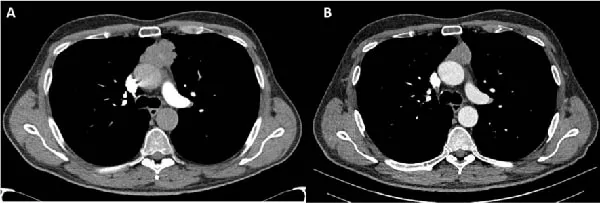
CT staging images before (A) and after (B) neoadjuvant therapy.

The 53‐year‐old patient initially presented with an unintentional weight loss of ~8 kg within 2 months and pronounced night sweats. The physical examination revealed no specific pathological findings; the patient was in good general health with no signs of acute illness. For further diagnosis, a CT scan was performed, revealing a suspicious mass measuring 43 × 33 × 52 mm^3^ in the anterior mediastinum without clear signs of local infiltration (Figure 1A). A CT‐guided biopsy of this mediastinal mass revealed a SCC of the thymus.

Following the verdict of an external tumor board, the patient was referred to our thoracic surgery department for evaluation of surgical resection. PET/CT imaging added to the staging revealed a high degree of suspicion of a limited left‐sided pleural carcinomatosis, leading to a diagnostic RATS with pleural biopsy, which confirmed the morphological suspicion of pleural carcinomatosis. Additionally, inspection of the tumor site raised suspicion of invasion into the pericardium and the innominate vein.

Based on the recommendation of our interdisciplinary tumor board, systemic therapy was initiated with four cycles of the PAC protocol (cisplatin, doxorubicin, and cyclophosphamide). Subsequent restaging showed an excellent morphological response to the therapy (Figure 1). Therefore, after consultation with the patient and the oncologists, an extended surgical resection of the tumor with simultaneous left‐sided pleurectomy, lymphadenectomy, and intraoperative HITOC was planned, considering the patient’s young age, lack of comorbidities, no infiltration of the ascending vessels, and the unilateral limited pleural involvement as an individualized treatment concept.

### 2.2. Intraoperative Procedure

**Figure 2:**
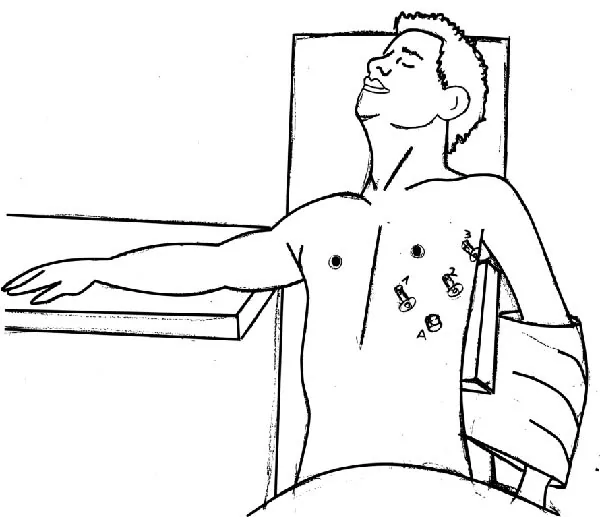
Author‐generated schematic illustration of patient positioning and trocar placement for left‐sided robotic‐assisted resection using the da Vinci Xi system. The patient was placed in a semi‐lateral position, with a wedge under the left thorax resulting in slight rotation toward the right side. The left arm was suspended in a sling to keep the left hemithorax unobstructed. Trocar configuration: camera trocar (2) in the fifth intercostal space at the midaxillary line, two additional robotic trocars in the third intercostal space at the midaxillary line (3) and in the sixth intercostal space at the anterior axillary line (1), as well as an assistant trocar in the eighth intercostal space, midway between the camera port and the left arm port (A).

In May 2024, we performed a left‐sided robotic‐assisted thoracoscopic extended en bloc thymus resection with partial pericardectomy, resection of the innominate vein, cytoreductive pleural surgery, mediastinal lymphadenectomy, and subsequent HITOC using the da Vinci Xi surgical system (Intuitive Surgical, Sunnyvale, CA, USA). The patient was positioned semi‐laterally for a left‐sided approach, with a wedge placed under the left hemithorax to achieve a slight rightward tilt and the left arm suspended in a sling to avoid obstruction of the operative field (see Figure 2). The camera port was inserted in the fifth intercostal space at the midaxillary line, followed by CO_2_ insufflation. Two additional robotic trocars were placed in the third intercostal space at the midaxillary line and in the sixth intercostal space at the anterior axillary line. The assistant trocar was placed in the eighth intercostal space, midway between the camera port and the anterior working port (see Figure 2).

Intraoperative exploration revealed three macroscopically visible lesions of the parietal pleura, which were resected with macroscopic safety margins (Supporting Information 1: Video 3). Two of the lesions were located on the left apical pleura and one on the dorsobasal parietal pleura. Histologically, however, only the sample from the dorsobasal pleura showed an infiltrate with a diameter of 30 mm, which is consistent with pleural carcinomatosis. To maximize cytoreduction, including removal of potentially occult pleural tumor deposits, parietal pleurectomy including mediastinal, costal, apical, and diaphragmatic pleurectomy was performed (Supporting Information 2: Video 5 and Supporting Information 3: Video 6). In addition, a metastasis‐suspicious lesion of the visceral pleura was identified on the lung surface and resected (Supporting Information 4: Video 4). As no pulmonary metastases had been detected during preoperative stage, no lung resection was required. A complete visceral pleurectomy was not performed because the anticipated risk of parenchymal injury was considered to outweigh the potential oncologic benefit.

After completion of the resection, drains were inserted through the four trocar sites to establish a closed HITOC circuit with two inflow lines, two outflow lines, and a temperature probe. The incisions around the drains were closed, and a test perfusion with saline solution was performed to confirm correct system function and the absence of leakage before administration of cisplatin‐based HITOC at 42°C for 60 min. The cisplatin dose was calculated individually based on the body surface area; in the present patient, 200 mg of cisplatin in 400 mL of saline solution was used. After completion of HITOC, two of the four drains were removed, and the corresponding access sites were closed. Two chest drains were left in place: one basally positioned drain for pleural fluid evacuation and one apically positioned drain for air evacuation. The procedure was completed without intraoperative complications. The individual surgical steps are presented in the supporting information videos:

1. DaVinci robot‐assisted left thoracoscopy (Supporting Information 5: Video 1).
2. Radical thymectomy en bloc with pericardium and innominate vein and mediastinal lymphadenectomy (Supporting Information 6: Video 2).
3. Radical resection of the localized parietal pleural metastasis (Supporting Information 1: Video 3).
4. Radical resection of the localized visceral pleural metastasis (Supporting Information 4: Video 4).
5. Mediastinal pleurectomy (Supporting Information 2: Video 5).
6. Parietal pleurectomy (Supporting Information 3: Video 6).

### 2.3. Postoperative Course

Postoperatively, the patient was extubated in the operating theater and transferred to our intensive care unit for cardiorespiratory monitoring and intensified pain management. On postoperative day (POD) 2, the patient was admitted to our regular ward in stable cardiovascular condition. His subsequent course was as expected. The patient recovered well from the operation and was easily mobilized with symptomatic analgesic therapy. The patient was fully mobilized by POD 3. No clinical or laboratory evidence of infection, postoperative bleeding, or renal dysfunction due to the platinum‐based HITOC was apparent. The two chest drains were removed on PODs 2 and 4. Postoperative X‐rays showed normal findings. The patient was discharged on POD 5.

The histological examination of the surgical specimen showed a major pathological response with the remains of a 2 mm, poorly differentiated SCC of the thymus with mediastinal fatty tissue infiltration and isolated foci of pleural carcinomatosis with a final tumor TNM stage ypT1b, ypN0 (0/3), ypM1 (pleura), RX, L0, V0, and Pn0; malignancy grade: G3, Masaoka–Koga stage IVa. Adjuvant radiotherapy of the thymic bed was initiated based on the recommended treatment concept of the ESMO guideline for thymic epithelial tumors [1].

Postoperative imaging surveillance consisted of routine chest radiographs during the in‐hospital course and cross‐sectional chest CT during the oncologic follow‐up. A chest CT performed 3 months after completion of adjuvant radiotherapy showed no evidence of tumor recurrence.

During follow‐up imaging in December 2025, ~19 months after surgery, a solitary 16 mm lesion in liver segment VII was detected and considered highly suspicious for metastatic disease. The case was rediscussed in the interdisciplinary tumor board, where surgical metastasectomy was recommended. The liver resection was subsequently performed in January 2026. Histopathological examination confirmed a 19 mm hepatic metastasis originating from the previously diagnosed thymic SCC. Importantly, no evidence of local thoracic recurrence or pleural relapse was detected. Following postoperative review in the multidisciplinary tumor board, continuation of regular oncological surveillance was recommended.

## 3. Discussion

This case report describes the first fully robotic‐assisted extended thymic carcinoma resection with pleurectomy and intraoperative HITOC. The current literature suggests that HITOC, in combination with cytoreductive surgery, is a promising treatment option for thymic malignancies with pleural spread [4, 5, 8, 9]. A retrospective analysis of 29 patients with thymic malignancies and pleural carcinomatosis from the Thoracic Surgery Department of the University Hospital Regensburg demonstrated a recurrence‐free 5‐year survival of 54% and an overall 5‐year survival rate of 80.1% [5].

The use of intraoperative chemotherapy, combined with significant surgical trauma, represents a substantial inflammatory trigger. Although case series have not shown an increased rate of postoperative circulatory or renal impairment [4, 5, 8, 9], HITOC remains a significant burden on the patient’s recovery. Minimally invasive surgery can potentially reduce surgical trauma and the systemic inflammatory response, leading to enhanced recovery and shorter hospital stays. This advantage is evident in the present case. While RATS and video‐assisted thoracoscopic surgery (VATS) appear to be equivalent in lung resections for lung carcinomas [10], RATS demonstrates superiority in the surgical treatment of thymic malignancies [11, 12].

It should be emphasized that most published series on cytoreductive surgery combined with HITHOC for pleural malignancies have been performed through open approaches. Open thoracotomy remains an important strategy, particularly in patients with extensive pleural disease or complex anatomical conditions because it provides wide exposure for pleural decortication, tumor resection, and intrathoracic perfusion. On the other hand, recent reviews have also highlighted the marked heterogeneity of indications, technical protocols, and chemotherapeutic regimens used for HITHOC, underlining the need for further standardization. In carefully selected patients with a limited unilateral pleural tumor burden, however, a minimally invasive robotic approach may offer relevant advantages by reducing access trauma while maintaining excellent visualization and instrument articulation for meticulous pleural inspection and cytoreduction. Therefore, our case should not be interpreted as an argument against open surgery but rather as proof of feasibility in a highly selected patient with limited pleural dissemination and favorable anatomy [13, 14].

Careful patient selection is likely a key prerequisite for the successful application of this strategy. In our patient, pleural disease was limited to a small number of unilateral pleural lesions without diffuse pleural rind formation or extensive visceral pleural involvement. Furthermore, neoadjuvant chemotherapy resulted in a marked radiological response, suggesting favorable tumor biology and increasing the likelihood of effective cytoreduction. In contrast, patients with extensive bilateral pleural dissemination, a bulky pleural tumor burden, or poor response to induction therapy may be better candidates for alternative treatment strategies.

However, the literature on robotic surgery combined with simultaneous intrathoracic hyperthermic chemotherapy remains very limited. Romano et al. [15] reported a retrospective series of nine consecutive patients with pleural recurrence of thymoma or early‐stage malignant pleural mesothelioma treated with robotic surgery followed by HITHOC; surgery consisted either of pleurectomy or removal of pleural lesions, and no intraoperative or renal complications related to perfusion were observed. To our knowledge, simultaneous robotic extended thymic carcinoma resection combined with unilateral cytoreductive pleurectomy and HITOC has not yet been reported. Our case therefore expands the currently available minimally invasive experience in this field [15].

A relevant limitation of this report is that the completeness of pleurectomy and the oncologic adequacy of resection of the visceral pleural lesion cannot be fully demonstrated by the video material alone. For this reason, the interpretation of the surgical videos must be combined with the operative report, histopathological findings, and postoperative imaging follow‐up. Further reports with standardized technical documentation and longer follow‐up are needed to better define the oncologic value of this approach.

Extended follow‐up of the present patient demonstrated the development of a solitary hepatic metastasis ~19 months after surgery, which was subsequently treated by surgical metastasectomy. Importantly, no evidence of local thoracic recurrence or pleural relapse was observed during this period. Although conclusions regarding oncological efficacy cannot be drawn from a single case, these findings may suggest durable local disease control following robotic cytoreductive surgery and HITOC. At the same time, the occurrence of distant metastatic progression highlights the aggressive biological behavior of advanced thymic carcinoma and underlines the importance of continued oncological surveillance.

Given the novelty and complexity of this approach, it is important to recognize that it represents an individualized treatment strategy tailored to carefully selected patients. In our case, favorable factors included limited unilateral pleural dissemination, excellent performance status, absence of major comorbidities, and a marked response to neoadjuvant chemotherapy. Further research is required to better identify patients who may benefit most from this aggressive multimodal treatment. Larger case series and prospective studies, such as the CHOICE trial [16], are needed to establish the long‐term oncological value of this approach and refine patient selection criteria.

## 4. Conclusion

RATS combined with HITOC appears to be a feasible and safe treatment option in carefully selected patients with locally advanced thymic carcinoma and limited pleural dissemination. This case report demonstrates that the procedure can lead to rapid postoperative recovery and minimal complications. However, given the individualized nature of this treatment approach, it is crucial to carefully select patients who are likely to benefit the most. Further studies are required to assess long‐term outcomes and to develop clear criteria for patient selection. Continued research and reporting of similar cases will be essential for establishing standardized protocols and optimizing patient outcomes.

## Author Contributions

Conceptualization: Joachim Schmidt and Donatas Zalepugas. Investigation: Maria Andrea Kröplin. Project administration: Maria Andrea Kröplin, Joachim Schmidt, and Donatas Zalepugas. Resources (provision and editing of the operation videos): Philipp Feodorovici and Donatas Zalepugas. Writing – original draft: Maria Andrea Kröplin, Hruy Menghesha, and Donatas Zalepugas. Writing – review and editing: Joachim Schmidt, Peter Keller, Nicolas Eckstein, and Jens Buermann.

## Funding

The authors have nothing to report. Open Access funding enabled and organized by Projekt DEAL.

## Consent

Written informed consent was obtained from the patient for publication of this case report and all accompanying images and supporting information.

## Conflicts of Interest

The authors declare no conflicts of interest.

## Supporting Information

Additional supporting information can be found online in the Supporting Information section.

## Supporting information


**Supporting Information 1** Video 3: Radical resection of the localized parietal pleural metastasis.

**Supporting Information 2** Video 5: Mediastinal pleurectomy.

**Supporting Information 3** Video 6: Parietal pleurectomy.

**Supporting Information 4** Video 4: Radical resection of the localized visceral pleural metastasis.

**Supporting Information 5** Video 1: DaVinci robot‐assisted left thoracoscopy.

**Supporting Information 6** Video 2: Radical thymectomy en bloc with pericardium and innominate vein and mediastinal lymphadenectomy.

## Data Availability

The data that support the findings of this study are available upon request from the corresponding author. The data are not publicly available due to privacy or ethical restrictions.
